# York County Hospital Inquiry

**Published:** 1913-05-03

**Authors:** Harriet Fawcett

**Affiliations:** 6 Pickering Terrace, York


### York County Hospital Inquiry.

THE SLIPPER CASE.

In connection with the verbatim report of the proceedings of the Special Commission appointed to inquire into the charges against the administration of this hospital made by Drs. Macqueen and Shepherd which appeared in The Hospital of March 15, the following letter from Mrs. Fawcett, the patient concerned, was published in the *Yorkshire Herald* of April 26, 1913. The evidence in connection with this case appears on pages 129 and 130 of The Hospital of 26th ult. The following is the letter :—

To the Editor of the *Yorkshire Herald*.

Sir,—Will you allow me, through the medium of your paper, to make a statement *re* my case in the York County Hospital ?

In the report of the inquiry it was stated that Mr. Fawcett was quite satisfied with the operation and had nothing to complain about except the fact that Mrs. Fawcett was taken to the secretary's office without slippers (a very trivial complaint indeed, I should say). Allow me to say that the hospital authorities knew quite well that Mr. Fawcett had, and did lay a more serious complaint other than that I was taken to the secretary's office without slippers. I also wish to state that the statements set forth in the report which was presented to the President of the inquiry and was shown to me by the secretary were not true. I further wish to make it clear that Nurse Sharp was not in any way to blame for my illness, and that, in my opinion, she was the most humane and thorough nurse that I came in contact with during my stay in the hospital. I only wish there were more like her, and I protest emphatically against any blame of my case being placed upon Nurse Sharp.—Yours truly,

6 Pickering Terrace, York. Harriet Fawcett.

